# Hypovitaminosis D is Independently Associated with Metabolic Syndrome in Obese Patients

**DOI:** 10.1371/journal.pone.0068689

**Published:** 2013-07-31

**Authors:** Ilaria Barchetta, Marzia De Bernardinis, Danila Capoccia, Marco Giorgio Baroni, Mario Fontana, Antonio Fraioli, Sergio Morini, Frida Leonetti, Maria Gisella Cavallo

**Affiliations:** 1 Department of Internal Medicine and Medical Specialties, Sapienza University of Rome, Rome, Italy; 2 Department of Medical Pathophysiology, Sapienza University of Rome, Rome, Italy; 3 Endocrinology and Metabolism, Department of Medical Sciences, University of Cagliari, Cagliari, Italy; 4 Human Anatomy, (CIR), University Campus Bio-Medico, Rome, Italy; John Hopkins Bloomberg School of Public Health, United States of America

## Abstract

**Background:**

Metabolic syndrome (MS) and hypovitaminosis D represent two of the most diffuse condition worldwide, reaching pandemic proportions in industrialized countries, and are both strongly associated with obesity. This study set out to evaluate the presence of an independent association between hypovitaminosis D and MS in an adult population of obese subjects with/without MS.

**Methods:**

We recruited 107 consecutive obese subjects, 61 with MS (age(mean±SD) 45.3±13.3 years, BMI(mean±SD): 43.1±8.3 kg/m^2^) and 46 without MS (age: 41.8±11.5, p = n.s., BMI:41.6±6.5 kg/m^2^, p = n.s.) comparable for sex, BMI, waist circumference and body fat mass, evaluated by bioimpedentiometry. 25(OH) vitamin D_3_ levels were measured by colorimetric method. Insulin resistance was estimated by fasting blood insulin, HOMA-IR and ISI.

**Results:**

Serum 25(OH)D_3_ levels were significantly lower in MS obese patients than in obese subjects without MS (median(range) 13.5(3.3–32) *vs* 17.4(5.1–37.4), p<0.007). Low 25(OH)D_3_ levels correlated with glycaemia (p<0.007), phosphate (p<0.03), PTH (p<0.003) and the MS (p<0.001). Multivariate model confirmed that low 25(OH)D_3_ levels were associated with the diagnosis of MS in obese patients independently from gender, age, serum PTH and body fat mass. After stratifying the study population according to 25(OH)D_3_ concentrations, patients in the lowest quartile showed a markedly increased prevalence of MS compared to those in the highest quartile (OR = 4.1, CI 1.2–13.7, p = 0.02).

**Conclusions:**

A powerful association exists between hypovitaminosis D and MS in obese patients independently from body fat mass and its clinical correlates. This indicates that the association between low 25(OH) D_3_ levels and MS is not merely induced by vitamin D deposition in fat tissue and reinforces the hypothesis that hypovitaminosis D represent a crucial independent determinant of MS.

## Introduction

Vitamin D is a lipophilic hormone synthesized in the skin by UV-mediated isomerization of 7-dehydrocholesterol and subsequently converted to active 1,25(OH)_2_D_3_ by two consecutive renal and hepatic hydroxylations. Besides its role in calcium-phosphate regulation and bone metabolism, it has a potential role in the development of insulin resistance-related conditions, such as obesity, type 2 diabetes mellitus (T2D), systemic hypertension and metabolic syndrome (MS) [1]–[4]. Vitamin D insufficiency is now considered to involve more than half the world’s population [5]–[6] and more than 75% of those with MS [7].

The nexus between hypovitaminosis D and obesity has been identified in the selective deposition of vitamin D, a lipophilic molecule, in subcutaneous and visceral adipose tissue [8]–[11].

BMI [12]–[18] and body fat [19]–[24] were inversely related to serum 25(OH) vitamin D in several studies. Blum M *et al.* found a positive correlation between serum and fat tissue 25(OH) vitamin D concentrations measured by liquid chromatography mass spectrometry in morbidly obese individuals [25].

Fat acts as a large capacity depot for the storage and release of vitamin D, accumulating vitamin D proportionally to its serum concentration and releasing it at a much slower rate, proportionally to the quantity of fat [8]. This may significantly affect 25(OH) vitamin D_3_ bioavailability and biological activity. Obese patients also show a reduced response of serum 25(OH) vitamin D_3_ levels to UV-B irradiation and to oral vitamin D administration compared with non-obese individuals [9].

Besides this evidence, it has been demonstrated that the visceral compartment volume, notoriously associated with the presence of MS, is more closely associated with vitamin D deficiency compared with subcutaneous fat, although the volume of the latter is more extensively represented in humans [23]. Furthermore, a number of studies found a strong association between low 25(OH) vitamin D_3_ levels and the diagnosis of MS and other dysmetabolic conditions [12]–[16]. Indeed, the fact that hypovitaminosis D is just an effect of vitamin D storage in fat tissue or a condition associated with insulin-resistance remains unclear [26].

Thus, the aim of this study was to test the hypothesis of an independent association between low 25(OH) vitamin D_3_ and MS in obese patients with and without MS. In particular, two groups of subjects with the same degree of obesity, selected based on the presence or absence of MS, were compared to determine the contribution of body fat mass in the association between vitamin D insufficiency and the development of metabolic abnormalities.

This is to our knowledge the first study designed to establish the role of body fat mass in the link between hypovitaminosis D and MS.

## Materials and Methods

### Population

We recruited 107 consecutive obese patients, 61 patients with a diagnosis of MS (30 patients with 3, 21 patients with 4 and 10 patients with 5 MS’ components) and 46 without MS, among subjects referring to the Endocrinology day-hospital of Sapienza University of Rome who underwent metabolic evaluation. The two groups were comparable for sex, age, BMI, waist circumference and body fat percentage, as evaluated by means of electric bioimpedentiometry (BIA), as shown in table 1. To be eligible for the study, patients had to fulfil the following criteria: age between 18 and 65 years, European extraction, BMI ≥30 kg/m^2^, informed consent, no history of cancer, liver/kidney failure or kidney stones, hypo-or primary hyperparathyroidism, sarcoidosis and bariatric surgery. None were taking vitamin D/calcium supplements or fortified-foods, bisphosphonates, estrogens or were affected by chronic diseases, physical limitations and other conditions that could negatively impact on 25(OH) vitamin D_3_ synthesis, such as immobilization or recent/frequent hospitalizations. In order to reduce the risk of possible bias in 25(OH) vitamin D3 measurement due to different latitude and global solar radiation on the ground, we selected only caucasian individuals living permanently in Rome at least during the six month before blood collection. In addition, all study participants underwent blood sampling in the same season (winter period). During the medical history collection we recalled our patients’ lifestyle and did not find significant differences among these obese subjects with regards of physically activity or time spent outdoor, as they all usually have a sedentary lifestyle.

**Table 1:** Clinical and biochemical characteristics of study population according to the presence (MS+) or absence (MS−) of MS. Student’s T test.

	MS+ (n = 61)	MS− (n = 46)	p-value
Sex (M/F)	34/27	25/21	n.s.∧
Age (years)	45.3±13.3	41.8±11.5	n.s.
BMI (kg/m^2^)	43.1±8.3	41.6±6.5	n.s.
Waist circumference (cm)	125.4±14.9	122.5±17.2	n.s.
Hip circumference (cm)	130.9±13.7	138.8±14.9	n.s.
Waist/Hip ratio	0.96±0.1	0.86±0.1	0.01
Body fat mass (%)	46.6(23.8–55.7)	48.1(31.9–59.4)	n.s.
SBP (mmHg)	130(110–180)	120(100–160)	<0.001
DBP (mmHg)	85(60–120)	80(60–80)	<0.001
Total cholesterol (mg/dl)	196.9±36.1	202.2±34.9	n.s.
HDL-cholesterol (mg/dl)	41(25–70)	53(31–92)	<0.001
LDL-cholesterol (mg/dl)	122.1±31.5	130.1±33.7	n.s.
Triglycerides (mg/dl)	147(48–454)	100.5(46–245)	n.s.
FBG (mg/dl)	109(75–268)	93(75–165)	<0.001
HbA1c % (mmol)	5.8(4.5–10.4)	5.5(4.5–7.8)	0.007
Fasting insulin (µU/ml)	36.8(8.1–107.3)	26.7(3.9–72.6)	<0.001
PTH (pg/ml)	37.4(17.2–297.2)	42.6(11.9–155)	n.s.
AST (UI/l)	23(11–131)	19(10–58)	0.02
ALT (UI/l)	30(4–140)	21(6–51)	0.001
HOMA-IR	9.1(1.5–37.6)	5.9(0.9–18.3)	<0.001
ISI	1.5(0.8–3.3)	2(0.9–8–8.4)	0.05
25(OH) vitamin D_3_ (ng/ml)	13.5(3.3–32)	17.4(5.1–37.4)	<0.007

∧ Chi-square test. Values are expressed as median(range) or mean±SD according to their distribution.

Pregnant/postpartum women were excluded. All subjects had a complete work-up including a clinical examination, anthropometric measurements and laboratory tests.

### Laboratory Determinations

Patients underwent fasting blood sampling to assess blood glucose (FBG, mg/dl), glycosylated hemoglobin (HbA1c, % - mmol/l), total cholesterol (mg/dl), high-density lipoprotein cholesterol (HDL, mg/dl), triglycerides (mg/dl), aspartate aminotransferase (AST, IU/l), alanine aminotransferase (ALT, IU/l), gamma-glutamyl transpeptidase (**γ**-GT, IU/l), serum calcium (mg/dl), ionized calcium (Ca^2+^, mmol/l) phosphorus (mg/dl), blood urea nitrogen (BUN, mg/dl) and creatinine (mg/dl) by standard laboratory methods. Insulin (IU/l) was measured by radio-immuno-assay (ADVIA Insulin Ready Pack 100, Bayer Diagnostics, Milan, Italy), with intra- and inter-assay coefficients of variation<5%. Serum parathyroid hormone (PTH, pg/ml) was measured by IRMA (N-tact PTHSP; Diasorin Inc.), with intra- and interassay coefficients of variation of 3 and 5.5%, respectively. Low-density lipoprotein (LDL, mg/dl) cholesterol value was obtained using Friedwald formula. MS was defined according to modified NCEP ATP-III criteria [27].

All subjects without a previous diagnosis of diabetes mellitus underwent standard oral glucose tolerance test (OGTT) with FBG and blood insulin measurement at baseline and 30, 60, 90 and 120 minutes after the ingestion of 75 grams of glucose; diabetes mellitus was diagnosed according to ADA 2009 criteria [28].

In order to assess the calcitriol balance in our population, we measured serum 25(OH) vitamin D_3_, the most stable circulating form of this molecule [29], [30]. Blood samples were obtained during the winter period and 25(OH) vitamin D_3_ was measured by a validated colorimetric method (LAISON, DiaSorin) on sera frozen immediately after separation and stored at −25°C for few days.

### Anthropometric Measurements

Weight and height were measured with patients wearing light clothing and no shoes. BMI was calculated as weight in kilograms divided by the square of the height in meters (kg/m^2^). Waist circumference (cm) was measured midway between the 12th rib and the iliac crest. Blood pressure (mmHg) was measured after five minutes of rest using an electronic auscultatory blood pressure recorder with an appropriately sized cuff based on the measurement of arm circumference with the patient sitting in the upright position. Three measurements were recorded, and the average of the second and third measurement was recorded and used in the analysis. Bioelectrical impedance measures were collected using the Body Composition Analyzer® (Tanita, TBF 300, Tanita Corp of America, Inc, Arlington Heights, Ill), which showed a high correlation with dual-energy x-ray absorptiometry in body fat estimation [31], after 12 hours of fasting and without taking liquids in the previous two hours, without clothing and/or metallic objects.

### Insulin Resistance Indexes

In order to provide an estimate of the degree of insulin resistance in study subjects, both static and dynamic insulin resistance indexes were calculated. The static indexes were: HOMA-IR [Homeostasis Model Assessment: FBG (mg/dl) * basal insulin (mg/dl)/22.5] and ISI Matsuda [Insulin Sensitivity Index: 10,000 √ FBG (mg/dl) * basal insulin (mg/dl) * (OGTT mean blood glucose * OGTT mean insulin)] [32].

### Statistics

All analysis were conducted by using SPSS version 17.0. Based on sample size calculation, 41 patients for each group were sufficient to reach the statistical power of 80% (alpha error 5%, confidence interval 95%). The variables not normally distributed were expressed by median(range) and underwent logarithmic transformation (log_10_) before statistical analysis; normal variables were expressed in the text and tables as mean ± standard deviation (SD). The comparison between averages of two independent groups was performed using Student's t test for continuous variables and chi-square was applied for dichotomic ones. Correlations were assessed by Pearson's coefficient. One-way analysis of variance (ANOVA) with the post-hoc Bonferroni test for multiple comparisons was used to determine if differences exist between means of patients belonging to different serum 25(OH) vitamin D_3_ quartiles. In order to identify clinical and biochemical predictors of serum 25(OH) vitamin D_3_ levels, we constructed a multivariate logistic regression model with selected variables having p-values<0.05 in univariate analysis (sex and age were also included). P-values<0.05 were considered statistically significant. The study was approved by the local Ethics Committee (Policlinico Umberto I, Rome) and was conducted in accordance with the principles of the Declaration of Helsinki. Written consent was obtained from all patients before the study.

## Results

Serum 25(OH) vitamin D_3_ levels were significantly reduced in obese patients with MS compared to obese subjects without MS, all comparable for sex, age, BMI, waist circumference and body fat mass percentage (13.5(3.3–32) ng/ml *vs* 17.4(5.1–37.4) ng/ml, p<0.007, respectively). This difference persisted also excluding patients affected by T2D (n = 26) from study population (14.4(5.7–32) ng/ml *vs* 17.4(5.1–37.4) ng/ml, p = 0.01, respectively).

Clinical and biochemical characteristics of study population in relation to the presence/absence of MS are shown in Table 1.

As expected, patients with MS had PAS, PAD, FBG, HbA1c and blood insulin significantly higher and HDL lower than subjects without MS. Reduction of insulin sensitivity as expressed by increased HOMA-IR and lower ISI was detected in MS patients compared with non-MS.

Serum 25(OH) vitamin D_3_ concentrations inversely correlated with FBG (Pearson’s coefficient: -0.26, p<0.007) serum phosphate (Pearson’s coefficient: −0.21, p<0.03) and PTH levels (Pearson’s coefficient: −0.28, p<0.003) but were not associated with anthropometrical parameters, fat mass percentage, the diagnosis of T2D and insulin resistance indexes in the univariate analysis.

Linear multivariate regression analysis demonstrated that low serum 25(OH) vitamin D_3_ levels are associated with the presence of MS independently from gender, age, BMI and serum PTH concentrations, as shown in Table 2.

**Table 2:** Multivariate linear regression analysis.

Model		Unstandardized Coefficients		Standardized Coefficients	t	Sig.
		B	Std. Error	Beta		
	MS	−4,316	1,547	−,290	−2,790	**,006**
	Age	,015	,064	,026	,243	,808
	Sex	,250	1,615	,016	,155	,877
	PTH	−10,693	3,658	−,288	−2,923	,004
	T2D	,299	1,806	,018	,166	,869

Relationship between serum 25(OH) vitamin D_3_ levels (dependent variable) and clinical-biochemical parameters in the study population. *Dependent Variable: 25(OH) vitamin D_3_*.

In order to describe the metabolic phenotype of study subjects according to their vitamin D status, our population was divided into quartiles in relation to serum 25(OH) vitamin D_3_ concentrations. Patients belonging to the lowest vitamin D quartile showed a markedly increased prevalence of MS with an OR of 4.1 (CI 1.2–13.7, p = 0.02) compared to those in the highest quartile.

Differences between vitamin D quartiles are showed in Table 3.

**Table 3:** Clinical and biochemical parameters of study population according with serum 25(OH)vitamin D_3_ quartiles. ANOVA test Bonferroni-adjusted.

25(OH)D_3_ (ng/ml)	I quartile 25(OH)D_3_≤11.6	II quartile 25(OH)D_3_ 11.7–15.8	III quartile 25(OH)D_3_ 15.9–21.3	IV quartile 25(OH)D_3_≥21.4	P- value*
N.	26	27	27	27	–
FBG (mg/dl)	113(81–228)	102(75–219)	94(76–218)	96(75–172)	0.01
PTH (pg/ml)	40(19–105)	40(16.2–97.8)	33.5(20.2–63)	36.9(11.9–58.9)	0.02
Phosphate(mg/dl)	3.6±0.6	3.3±0.4	3.4±0.5	3.2±0.4	0.04
Fat mass (%)	47.7(23.8–55.7)	45.6(27.4–55.2)	47.8(31–59.4)	47.9(34.3–54.9)	n.s.
BMI (kg/m^2^)	42.4±8.9	44.9±7.6	41.8±8	42±4.9	n.s.
MS+ % (n)	77% (20)	58% (15)	55.5% (15)	40.7% (11)	0.01∧

* Comparison between high and low quartile.

∧ Chi-square test applied. Values are expressed as median(range) or mean±SD according to their distribution.

## Discussion

In this study we demonstrated that serum 25(OH) vitamin D_3_ levels are significantly lower in obese patients affected by MS than in obese subjects without MS and comparable for sex, age, BMI, waist circumference and body fat mass. The association between low serum 25(OH) vitamin D_3_ concentration and the diagnosis of MS was independent from PTH levels and the presence of T2D. The existence of independent association between hypovitaminosis D and dysmetabolic conditions such as MS, T2D, hypertension and liver steatosis has been demonstrated in several studies [1]–[4] and, more speculatively, hypovitaminosis D has been hypothesized as a primary cause of obesity [23]. However, the potential cause-effect relationship between the presence of low 25(OH) vitamin D_3_ levels and obesity/obesity-related conditions is still debated. Since BMI and fat mass have been demonstrated to inversely correlate with 25(OH) vitamin D_3_ concentrations, the vitamin D specific metabolism may account for this association and has been proposed as the cause of hypovitaminosis D in obese people [32]. Thus vitamin D, being a lipophilic hormone, is physiologically sequestered and stored in adipose tissue leading to reduced serum bioavailability [8]. Other investigators suggested additional potential reasons to explain the contribution of obesity to hypovitaminosis D such as reduced time spent out of door, resulting in an insufficient sun exposure and reduced skin vitamin D_3_ synthesis, and exposure of smaller skin areas, reducing UV-rays penetration [33]–[34]. Other studies have reported an association between dysmetabolic conditions and hypovitaminosis D independently from adiposity [12]–[16]. In addition, low 25(OH) vitamin D_3_ concentrations are associated with increased cardiovascular risk independently from traditional risk factors as shown in recent studies [35]–[37]. In this study we demonstrated that in obese patients the presence of MS is the determinant of low serum 25(OH) vitamin D_3_ levels independently from the fat mass percentage and its correlates. Studies *in vivo* and *in vitro* demonstrated that the active form of vitamin D_3_ exerts an insulin-sensitizing action by increasing the expression of insulin receptors in peripheral tissues and facilitating insulin-mediated glucose transport. In addition, vitamin D_3_ directly regulates the free fatty acids (FFA) metabolism acting on the PPAR-γ and improves insulin resistance induced by FFA *in vitro*. Therefore, vitamin D deficiency may worsen the degree of insulin resistance by means of a higher FFA flow into the bloodstream [38]. In line with our results, Cheng S. *et al.* observed in non obese subjects that the visceral compartment volume, notoriously associated with the presence of MS, is more closely associated with vitamin D deficiency compared with subcutaneous fat, although the volume of the latter is more extensively represented in humans [23]. Although our study design does not allow to establish a causal relationship between low 25(OH) vitamin D_3_ levels and MS, data indicate that hypovitaminosis D in MS patients is not merely induced by vitamin D depot in adipose tissue since the reduction of 25(OH) vitamin D_3_ in obese patients without MS is significantly lower compared to what observed in obese patients with MS and this difference should account for the excess metabolic MS observed in obese patients in the lower range of vitamin D circulating levels. Consequently, it is reasonable to postulate that 25(OH) vitamin D_3_ levels may be a determinant of MS development in patients with obesity. On the other hand, MS and insulin-resistance could represent the “first hit” able to worsen vitamin D balance by interfering with its metabolism and/or action on target tissues. In conclusion, reduced serum 25(OH) vitamin D_3_ concentrations represent a determinant of MS in obese patients. The insulin-sensitizing action of vitamin D rather than the distribution volume of this hormone is likely to be responsible for the thigh association between hypovitaminosis D and dismetabolic conditions.
